# Supplementary material for: Glomeromycota associations with bamboos (Bambusoideae) worldwide, a qualitative systematic review of a promising symbiosis
Source: PeerJ. 2023 Nov 9;11:e16151. doi: 10.7717/peerj.16151 (PMC10640841; doi:10.7717/peerj.16151)
Supplement: Supplemental Information 1 [file peerj-11-16151-s001.docx]

Other

**Glomeromycota associations with bamboos (Bambusoideae) worldwide, a qualitative systematic review of a promising symbiosis**

Systematic Review and/or Meta-Analysis Rationale

Rationale for conducting the systematic review / meta-analysis

The need for sustainable crop and soil management including biological strategies is now well known. Arbuscular mycorrhizae are mutualistic symbioses of plants with soil fungi that play a key role in nutrient cycling in different agroecosystems and are known to form in plants of the family Bambusoideae, including the genus Guadua. However, scientific reports of this association are scarce and restricted to the Asian continent. We are motivated by the need to have as much information as possible on the fungi reported on this group of plants, including the current classification of symbionts. The need to promote research and publish results on the American continent, which has an important diversity of woody and herbaceous bamboo and arbuscular mycorrhizal fungi, is also highlighted.

Contribution that it makes to knowledge in light of previously published related reports, including other meta-analyses and systematic reviews

The paper presents the current state of knowledge regarding the symbiosis between Glomeromycota and Bambusoideae, based on information published in indexed scientific Journals while reflecting on the challenges and scarcity of research on this promising association found throughout the world. The reported relationship, based on up-to-date taxonomy, of bamboo and Glomeromycota provides an important and up-to-date reference document for the public interested in the two groups of organisms, researchers, academics, scientific societies, governmental entities as well as bamboo producers.
